# Real-world experience with colorectal cancer chemotherapies: patient web forum analysis

**DOI:** 10.3332/ecancer.2013.361

**Published:** 2013-10-10

**Authors:** Kathleen Beusterien, Sarah Tsay, Shadi Gholizadeh, Yun Su

**Affiliations:** 1 Outcomes Research Strategies in Health, Washington DC, 20008, USA; 2 Oxford Outcomes, Bethesda, MD, 20814 USA; 3 Bristol-Myers Squibb, Lawrenceville, NJ, 08648 USA

**Keywords:** colorectal cancer, internet, chemotherapy, social media

## Abstract

**Background:**

In contrast to clinical trials, patient web forums provide a unique opportunity for patients to spontaneously post their experiences and thoughts about diseases and treatments. This study explored the impact of colorectal cancer (CRC) treatments in these forums.

**Methods:**

This was a systematic cross-sectional qualitative analysis. Two active CRC web forums were identified based on four criteria: active for ≥five years, >12,000 total posts, >20 individuals currently browsing, and ≥10 new posts/day. All relevant threads (set of messages focusing on a topic) relating to treatment posted in July and December 2010 and February to March 2011 were reviewed and coded using MaxQDA software. A content analysis was performed identifying key themes.

**Results:**

The threads included 1522 posts by 264 individuals. Demographics were identified for 83% of the posters. Of these, 83% were CRC patients and 17% were family members; 76% were females, and the mean patient age was 49 years. The majority had advanced cancer (44% stage IV or metastatic, 40% stage III). The most common themes were side effects (62.3% of posts), treatment response (13%), and impact on personal, social, and work lives, and emotional distress (23.9%). The posters came to the online forums to have an emotional outlet, share experience, and seek advice. The emotional impacts primarily exemplified resilience and positive coping strategies. Formal knowledge regarding the likelihood of treatment response, magnitude of benefit, or side effects was lacking, which lead to uncertainty and anxiety. However, patients expressed appreciation for the availability of treatment options and the hope they provide.

**Conclusion:**

Online CRC communities provide patients with convenient and valuable emotional support and disease information. CRC and treatments may have profound impacts beyond efficacy and toxicity. Systematic information and decision tools may help to minimise uncertainties and help patients manage expectations and emotional distress.

## Background

Colorectal cancer (CRC) is a major health burden worldwide. Although the incidence and mortality from colon cancer have been declining in the United States, colon cancer remained the third most common cause of cancer-related mortality in 2008 [1]. Specifically, there were over 1.2 million new cancer cases and 608,700 deaths estimated to have occurred in 2008 [2]. The highest incidence rates are found in Australia and New Zealand, Europe, and North America [2]. The stage at diagnosis and interval to recurrence are the two factors most highly related to survival [3]. 

While the standard treatment option has been 5-fluorouracil (5-FU) chemotherapy alone for colon cancer that has advanced beyond the point of curative surgery, there has been a surge of additional treatment options, including combination chemotherapies, and, most recently, targeted biologic therapies [4]. New agents that have become available include cytotoxic agents such as irinotecan and oxaliplatin [5], oral fluoropyrimidines (capecitabine and tegafur), and biologic agents such as bevacizumab, cetuximab, and panitumumab [6]. The increasingly accepted paradigm of treatment for metastatic CRC is the continuum of care approach, where chemotherapy is individualised to patients’ needs. For example, the continuum of care approach allows for switching chemotherapies before the disease has progressed, taking breaks from treatment, combining various treatments, and surgical treatments in some cases [4]. 

Such individualised treatment in CRC makes the internet a valuable resource for evaluating the experience of patients with CRC. The internet, and, more specifically, patient blogs and forums, provides a rich source of data from which researchers can obtain patients’ thoughts and representations on their illness and treatment impacts. As early as 1998, researchers identified the internet as a burgeoning venue for cancer support groups [7]. In one of the first forays into the role of the internet in cancer support, Klemm and colleagues analysed 300 messages posted by members of an internet cancer support group, and they classified them into key categories including information giving/seeking, personal opinions, and encouragement/support.

Given that the internet has become a part of the everyday life of many people, it is not surprising that it has been discovered as a tool to use for research; this approach has been called ‘virtual ethnography’ [8]. Typically, data collection about patient experiences is limited to controlled clinical trials where patients may not feel comfortable or feel it a priority to discuss various impacts of their treatment or be confined to topics limited by a structured questionnaire or interview. Patient web forums provide a unique opportunity for patients to post their experiences and thoughts about diseases and treatments spontaneously. To the authors’ knowledge, there have been no web forum analyses in the published literature specific to CRC. The objective of the present study was to better understand patient experience with CRC chemotherapies in the real-world setting.

## Methods

This was a cross-sectional qualitative analysis focusing on CRC web forums. This research followed three main steps: (1) selection of web forums, (2) selection of threads, and (3) qualitative analysis of the threads. For the first step, the search for relevant CRC web forums was performed online via both Google and Yahoo search engines, which were selected given their wide use, as the results using different search engines may be different. Search terms included different combinations of disease terms, specifically ‘colorectal cancer’, ‘colon cancer’, and ‘metastatic colorectal cancer’, and ‘patient,’ ‘support’, ‘blog’, and/or ‘forum’. This search was conducted by two independent researchers using two different computers, as search findings may be dependent on previous searches by the computer user. The previous two steps were repeated every other day for one week to account for websites being down for repair or other maintenance issues. The two most active CRC web forums were identified for inclusion in the analysis based on four criteria: site active for >5 years, >12,000 total posts on the forum, >20 individuals currently browsing, and >10 new posts/day.

The second research step was the selection of threads within the two CRC web forums. A thread refers to the group of messages that are posted in response to a message or topic. To help ensure the robustness of the findings, any thread with a post related to experience with chemotherapy was reviewed in the analysis. Because the forums were organised by the date of last comment, threads occurring between specific dates were reviewed. Specifically, all threads relating to treatment experience posted between specified randomly selected dates were abstracted for review: Website 1: 23 February 2011 to 07 March 2011; Website 2: 1 July 2010 to 31 July 2010, 1 December 2010 to 7 December 2010, 17 February 2011, and 28 February 2011.

In the third step, the complete threads were copied from the forums into documents that were uploaded onto the qualitative data analysis software MaxQDA (Berlin, Germany). Within MaxQDA, all posts within each abstracted thread were reviewed. All posts meeting the following eligibility criteria were coded (assigned a descriptive label) for analysis: (1) post referred to an impact associated with chemotherapy, (2) poster discussed the personal impact associated with chemotherapy or the impact on a family member or friend, and (3) post was in English. The researchers met daily to discuss themes and developing a coding (structure) system that served as the basis for organisation and evaluation of content. The researchers coded any post referring to an impact of chemotherapy; this could either be a side effect or a functional impact. Side effects were coded using their names, and functional impacts were coded broadly based on the functional area, such as activities of daily living, work, or emotional impacts. Three researchers each coded a subset of threads, and one researcher reviewed and confirmed all code assignments.

## Results

### Population characteristics

**Figure 1.**
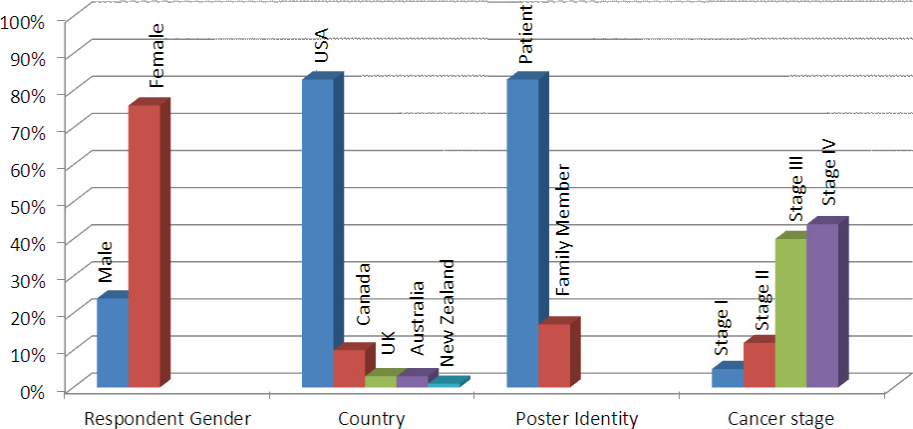
Available sample demographics (*N* = 264 posters).

During the aggregate 52-day period in which the web forums were reviewed, a total of 135 threads (groups of messages posted on a topic) were eligible to be included in the analysis, as they referred to experience on chemotherapy for CRC. The threads included a total of 1522 posts by 264 individuals. In the signature line of 83% of the posters, demographic and/or tumour information were listed. Of these, 83% were CRC patients, and 17% were family members; 76% were females, and the mean age of the patients was 49 ± 11 (range: 23–79) years. The majority had advanced cancer (44% stage IV or metastatic, 40% stage III) (Figure 1).

### Key concerns

A total of 918 codes were assigned; of these, 588 (64%) were side effects, and 330 (36%) were more distal impacts, such as work productivity, social, and emotional impacts. Table 1 reports the side effects that represented at least 2% of the side effect codes, and Table 2 lists example quotes for selected side effects. The severity of side effects was highly variable, ranging from those that were mild or that could be ameliorated with simple over-the-counter treatments, to those that had substantial impacts on patients’ daily lives.

Among the side effects, the most common were gastrointestinal issues (20.9%), which included descriptions of discomfort like having a ‘sour stomach’, ‘stomach cramps’, ‘gut pain’, ‘hard on GI tract’, ‘dull knots’, ‘burning’ as well as citing specific symptoms, including diarrhoea, constipation, nausea, and vomiting. The second most common side effect was skin problems (14.5%), which included rash, itchiness, hand-foot syndrome, and skin discoloration. Rashes were described as itchy, sore, measle-like, hive-like, and painful. Hand-foot syndrome was described as dryness, swelling, blistering, and peeling soles of the feet and palms. Another common skin problem was general allergic reaction to treatment. These reactions manifested themselves as rashes, blisters, and breakouts during or immediately after infusion.

Neuropathy was discussed in 11.2% of the side effect codes; the severity ranged from mild to debilitating: ‘weird sensation’, ‘a nuisance’, ‘frustrating’, ‘awful feelings in my feet and hands’, and ‘unbelievable pain’. Many with neuropathy expressed that the nerve damage and respective symptoms could be longstanding, and thus this was a particularly bad side effect to experience. Mouth problems were reported in 9.7% of the side effect codes. These included dry mouth, jaw pain, mouth sores, which could be painful and interfered with eating, change in taste, and dental issues, which included wearing down of teeth and fillings, gum problems, tooth sensitivity, and tooth loss. Other less frequent side effects included bodily pain/aches, fatigue, sensitivity to temperature, chemo brain (memory issues/dizziness), and blood issues, such as bleeding or bruising.

Table 3 lists the physical functioning and emotional impact codes (*N *= 330). These included treatment impacts on daily activities, including eating, dressing, and mobility (12.4%). In addition, both chemotherapy administration (13.6%) and treatment schedule (5.8%) were reported as disruptive to performing activities. For example, posters expressed issues with inconvenience of treatments, such as the necessity of carrying around the portable pump, and timing of treatments, including taking intervals out of one’s workday to go into a hospital to get infused. Some posters expressed a preference for taking a pill as opposed to undergoing an infusion. Other activities impacted by CRC treatments include work, physical, and social activities.

Numerous emotional impacts were associated with treatment (Table 3). The emotional impacts that posters described were complex and, quite often, appeared to exemplify resilience and coping strategies. Specifically, 12.7% of the 330 physical and emotional codes focused on hope or appreciation for treatment: ‘Honestly, if it gave me a 1% higher chance of survival rate then I would have done it’. Many posters would reiterate slogans popular among cancer survivors such as the importance of being a ‘cancer warrior’, particularly in dealing with the adverse effects of treatments. The second most common emotional concept discussed was anxiety (8.8%). Many of these posts were mixed with respect to the focus of their anxiety—while being on treatment, or treatment side effects, may be anxiety-provoking, not being on a treatment that may be effective, or the possibility of having to discontinue treatment was more important to posters and could cause much higher anxiety.

**Figure 2.**
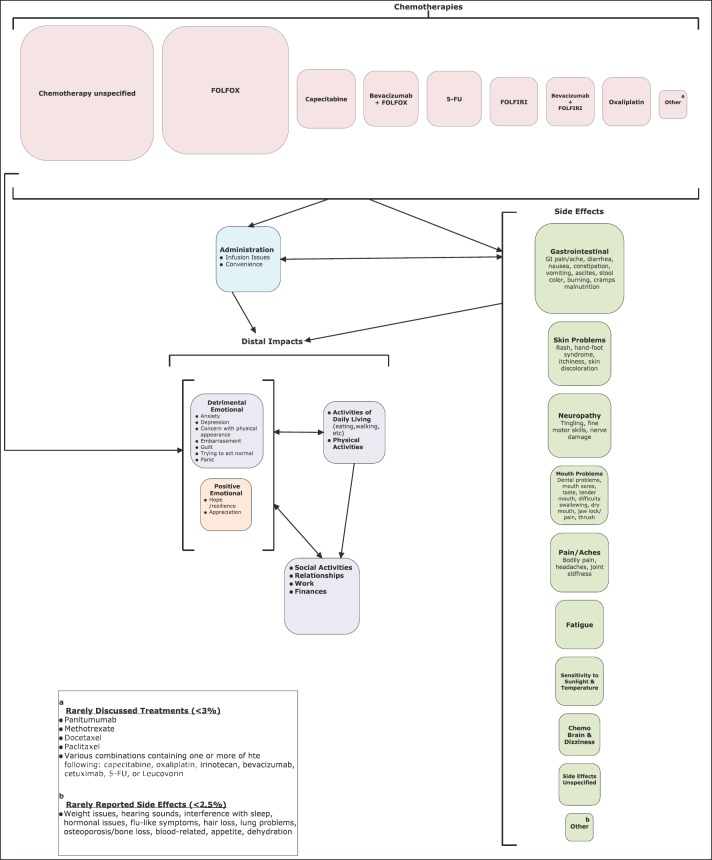
Conceptual model of real-world treatment experience among patients with CRC: the one-way arrows represent a one-way relationship where one effect causes the other, and the two-way arrows represent that the effects can cause one another; the sizes of each of the parameters (bubbles) represent the frequency with which they were mentioned.

The research findings are depicted in a conceptual model (Figure 2) that shows hypothesised relationships among treatments, side effects, and distal impacts. The one-way arrows represent a one way relationship where one effect causes the other, and two-way arrows represent that the effects can cause one another. The sizes of each of the parameters (bubbles) represent the frequency to which they were mentioned. For example, FOLFOX was the most often mentioned treatment; GI problems were more than twice as frequently mentioned as chemo brain/dizziness. From this conceptual model, it is shown that chemotherapy may have direct emotional impacts, both negative and positive, or may have distal impacts indirectly via administration issues, such as disruptions because of scheduling or infusion-related events and side effects.

## Discussion

The findings from this research provide a comprehensive picture of real-world experience with CRC treatments from the patient perspective. The web outcome analysis approach utilised in this study was a useful methodology to understand the key topics surrounding treatments prevalent in the discourse of CRC patients. Further, the findings, particularly as depicted in the conceptual model, can serve as useful tool in communications with patients about their concerns and illustrating for patients the impacts of treatments, both negative and positive, among their peers.

While in the clinical world today, there is increasingly more concern about toxicities of new treatments being introduced in CRC, in addition to efficacy concerns, the emotional benefits that treatment provides is an important area of consideration for patients, their families, and quality of life researchers. Specifically, based on this analysis, patients primarily are concerned with battling their cancer, and treatment provides them with the possibility of living longer and enjoying a normal life after the treatment. Although side effects from treatment may be anxiety-provoking, what is ‘top of mind’ for patients are concerns about whether or not treatment is working and having access to treatment. Patients indicated that even a small improvement in survival is of great importance from their perspective.

The psychological distress that comes with cancer diagnosis should not be underestimated. There is much research describing the psychological distress that is prevalent among cancer survivors [9–12]. Deimling and colleagues [12] developed a conceptual model based on Lazarus and Folkman’s (1984) [13] general stress model that shows cancer-related stressors, non-cancer stressors, and temporal factors relating to psychological distress that cancer survivors experience. Such work reinforces the finding from this research that treatment availability and effectiveness may have a substantial impact on emotional functioning.

As evidenced by the high level of activity in the forums, both between patients and their family members, participation and involvement in these forums appear to serve several purposes. First, the forums are a way to garner and share information pertaining to specific treatments and outcomes. Although posters likely can arrive at this information by way of more formal channels, such as educational media (books, online medical resource sites, medical articles, pamphlets) or enquiry to a medical professional, the high level of contribution and involvement in the forums exhibits that poster’s value one another’s opinions and experiences and are curious to learn if their experiences on treatment are in line with what others on similar treatments have experienced. Second, the forums are a source of support. Patients and their family members are extremely supportive of one another and often seem to follow other poster’s stories and treatment trajectories, providing positive reinforcement and words of encouragement quite commonly. Such social support may impact outcomes among patients. Indeed, a recent study found that patients’ living alone more often had synchronous metastases at presentation and were less often treated with combination chemotherapy than those cohabitating [14]. 

The methodology used in this research fills a gap in the literature and can be used as model for conducting internet-based research. While physicians can provide input into understanding the impacts of the disease, it is commonly accepted in patient-reported outcome research that the best way to understand the impacts of disease and treatment is from the patients themselves, and internet outcome analyses represent an efficient and comprehensive source to obtain this input. While a 1998 article examining internet support groups targeted for cancer survivors was titled ‘A *Nontraditional *Cancer Support Group: The Internet’ [7], in the years since, internet-based support groups have burgeoned. Researchers have studied this trend, although not specifically for CRC. For example, the internet was used by Hadert and Rodham [15] to explore patient experiences in arthritis and by Burri, Baujard, and Etter [16] to explore discussion in an online forum of ex-smokers. Zrebiec and Jacobson (2001) established and evaluated a support forum for diabetes patients and found it to be a useful strategy for engaging patients with chronic disease for emotional support and information exchange [17].

Factors that contribute to the popularity of online support forums for individuals with various health conditions have been described to include convenience, anonymity, and volume of online information [18]. Another factor, specifically for advanced CRC patients, may be that poor health prohibits visiting ‘live’ support groups. Indeed, as our analysis showed, the majority of the posters were at a later stage in disease. The present web analysis provided an opportunity to evaluate comments based on the ‘real world’ setting that can supplement traditional PRO collection, such as in a trial setting or other observational studies using standardised questionnaires or interview guides, where patients may be limited to responding to already established questions.

Compared with more traditional qualitative analyses that involve identifying key themes in interviews with individuals, the two web forums in this analysis were already organised by topic, and thus one could suggest that the information about the topics were already saturated. Specifically, all relevant comments related to a topic appear together, and it could be assumed that the posters have provided all the relevant information that they wish to on the respective topic. As such, we believe that our data were relatively comprehensive with respect to the concerns discussed on each topic.

This study has limitations. Given the source of the data, we cannot verify that the posters are actually on the treatments that they report. However, we would hypothesise that posters who are pretending to have such experiences would occur only minimally. Another limitation is that complete information with respect to cancer stage and other potential explanatory variables was not available for the complete sample. Third, while traditional modes of qualitative research with patients, such as focus groups and interviews, allow interviewers to probe, this was a retrospective analysis of web patient-reported outcomes for which the researcher is an outside observer. However, in many ways, the forums serve as a large focus group, allowing interactions among the entire community of posters, and thus they act as probes for one another. But there may be bias introduced due to the posters that were identified in this evaluation, as this likely is a group who enjoys blogging, and it is unknown how this may have impacted the findings. Finally, given that the source of data was the internet, the sample may be skewed where a disproportionate number may be more highly educated or have higher socioeconomic status than the general population of CRC patients.

## Conclusion

The present study explored the impact of CRC treatments via patient web forums, providing valuable input characterising the CRC patient population in a way that has not previously been explored. The descriptions of side effects reported by patients enable a more thorough interpretation of adverse events typically presented in the clinical literature. On balance, although patients reported difficulties being on treatment, they also expressed resilience and appreciation for the availability of treatment options and the hope they provide. This study adds to the limited existing qualitative CRC literature and increases our understanding of the impacts of CRC on patients’ lives.

## Conflicts of interest

Bristol Myers Squibb (BMS) sponsored this research. During this research, KB, ST, and SG worked for Oxford Outcomes, Inc., which provides consulting services to BMS. YS is an employee of BMS.

## Authors’ contributions

KB, ST, and SG conceptualised the study design, collected and analyzed the data, and developed the initial draft of the manuscript. YS assisted in study design and interpretation of the data and provided substantive comments on the manuscript draft.

## Figures and Tables

**Table 1.** Most frequently reported side effects (>2%) .

Side effect codes	*N *	*N* = 588 (%)
GI problems (diarrhoea, stomach pain, nausea, etc.)	123	20.9%
Skin problems (rash, itchiness, acne, etc.)	85	14.5%
Neuropathy	66	11.2%
Mouth problems (jaw pain, sores, dental problems)	57	9.7%
Bodily pain/headache	48	8.2%
Fatigue/tiredness	35	6.0%
Temperature-related issues (sensitivity, etc.)	34	5.8%
Chemo brain and dizziness	29	4.9%
Side effects unspecified	25	4.3%
Blood issues (bleeding, bruising, blood clot, etc.)	13	2.2%

**Table 2.** Example descriptions of selected side effects.

Side effect	Example quotes
GI problems	• My first couple of rounds was a nightmare, with 11 days in a row of the big D. • I have had gut pain with every round. In the beginning, I kept thinking that it was cancer spreading, but I now realise it is the chemo. After this last round, I actually went to the ER because I was having such bad pain. It is so hard on the GI tract.
Skin problems	• I shave my hair right away before chemo. I do it right away because if you wait too long, you get the rash on your scalp and then you have to be very careful when you shave it because it is sore and itchy. • My palms and the soles of my feet turned pitch black while I was on treatment. It was horrid. I would be embarrassed to hand other people things. And I remember going to the pool. I got the worst stares from people. It was awful.
Neuropathy	• My fingers still tingle. It is hard to do small things like button shirts or tie fishing knots. My feet feel slushy. • I seem to want to fall down a lot, and my oncologist said it is because the treatment has destroyed the sensory nerves that we have in our limbs.
Mouth sores/ dental problems	• I remember those horrible mouth sores and mouth dryness. • The other day a tooth broke in half, and the one next to it is, for lack of better words, crumbling. • I went to the dentist and was told the enamel on my teeth had probably been eaten away by chemo.
Bodily pain	• Extreme back pain episodes that are sporadic and begin about 30 hours after the infusion and cease within about 12 hours after onset.
Fatigue	• Fatigue is a huge side effect of cancer treatment, and there is nothing better than closing your eyes for a short time to rejuvenate ourselves and our outlook
Chemo brain	• I also had chemo brain. I could not remember the correct word that I was searching for when describing something. I still have it, but not to the degree as it was many months ago. It was frightening.

**Table 3.** Physical functioning and emotional impacts reported in CRC web forums.

Physical and emotional well-being impacts	*N *	*N* = 330 (%)
Activities of daily life (eating, dressing, walking, etc.)	41	12.4
Activity disruption due to chemotherapy administration	45	13.6
Activity disruption due to treatment schedule	19	5.8
Work	41	12.4
Interpersonal relationships	23	7.0
Physical activities	18	5.5
Social activities	17	5.2
Sexual functioning	1	0.3
*Emotional impacts *		
Hope/appreciation	42	12.7
Anxiety	29	8.8
Depression	22	6.7
Emotional	15	4.5
Concern with physical appearance	7	2.1
Anxiety from carcinoembryonic antigen (CEA) testing	6	1.8
Guilt	2	0.6
Trying to act normal	1	0.3
Embarrassment	1	0.3
